# Relationship stress at workplace was associated with problem drinking patterns: a nationwide Internet-based prospective cohort study

**DOI:** 10.1265/ehpm.26-00131

**Published:** 2026-09-19

**Authors:** Mami Wakabayashi, Hiroyasu Iso, Takahiro Tabuchi

**Affiliations:** 1 Kyushu University Institute for Asian and Oceanian Studies, Fukuoka 819-0395, Japan; 2 Institute for Global Health Policy Research, Bureau of Global Health Cooperation, Japan Institute for Health Security, Tokyo 162-8655, Japan; 3 Division of Epidemiology, School of Public Health, Tohoku University Graduate School of Medicine, Sendai 980-8575, Japan

**Keywords:** Alcohol Use Disorders Identification Test (AUDIT), Work-related stress, Brief Job Stress Questionnaire (BJSQ), Problem drinking, Interpersonal stress

## Abstract

**Background:**

Previous studies have limited longitudinal data with inconsistent relationships between workplace stress and problem drinking. This study aimed to investigate the association between work-related stress and the development of problem drinking among Japanese workers.

**Methods:**

A cohort study was conducted using data from a nationwide internet survey in Japan in 2022 and 2023. A total of 8,157 participants (55% men; mean age: 47.2 years) were categorized based on their Alcohol Use Disorders Identification Test (AUDIT) scores into non-problem drinking (0–7) and problem drinking (8–40). Work-related stress was assessed using the New Brief Job Stress Questionnaire (BJSQ), which includes 17 items across nine work-related stresses categories such as relationship stress, job demand (quantity and quality), physical demand, environmental stress, job control, skills utilization, suitability, and satisfaction.

**Results:**

From 2022 to 2023, 499 participants (360 males, 139 females) transitioned from non-problem to problem drinking. After adjusting for sociodemographic factors, living conditions, self-rated health, smoking and nine work-related strass mutually, male individuals with high relationship stress were more likely to develop problem drinking (adjusted OR: 1.77; 95% CI: 1.20–2.61), and similar but marginal association was observed in female (adjusted OR: 1.74; 95% CI: 1.00–3.04). In addition, male individuals with high physical demand were more likely to develop problem drinking (adjusted OR: 1.39; 95% CI: 1.00–1.94), but not in female.

**Conclusion:**

Among nine work-related stresses categories, high relationship stress at the workplace in male and female and high physical demand in male was associated with an increased risk of developing problem drinking.

**Supplementary information:**

The online version contains supplementary material available at https://doi.org/10.1265/ehpm.26-00131.

## Graphical Abstract

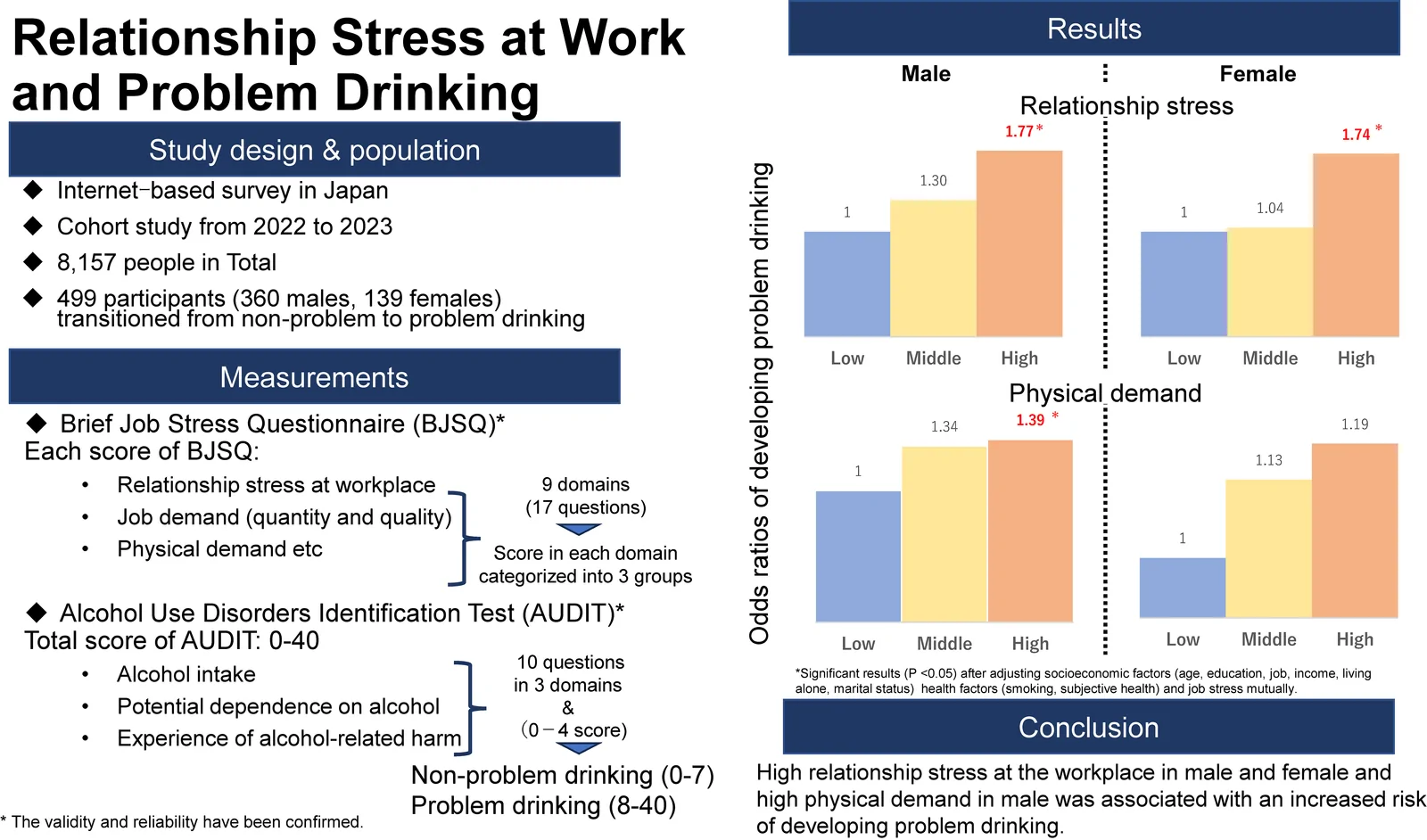

## 1. Introduction

Alcohol is the most important risk factor for disability and premature death in the world [1, 2]. Alcohol use was the seventh leading risk factor for both deaths and DALYs, accounting for 2.2% female deaths and 6.8% male deaths globally according to the analysis for Global Burden of Disease Study 2016 [3]. Although the negative impact of health, the detrimental effects of alcohol consumption on the health of populations are often downplayed or overwhelmed with widespread acceptance of alcohol use and social norms that support drinking behavior, and light to moderate alcohol consumption may help coping stress [2].

The most significant direct costs in employees who have problem drinking can undermine a workforce’s overall health and productivity lost such as absence from work, poor performance on the job, loss of job, and accidents resulting in death or disability. The existence of occupational subcultures that share values and expectations with alcohol drinking has also been noted [4]. In addition, some of workers drink to cope with stress or to overcome negative emotions arising from the physical and psychosocial factors in the work-environment [5]. It is necessary to better understand the factors underlying employees’ problem drinking.

The two major theoretical models of work stress on health were developed, the demand-control model and the effort-reward imbalance model [6]. The result between work strain and problem drinking such as alcoholism were shown to be inconsistent [6]. Some research showed positive associations between workplace stressors and harmful alcohol consumption in cross-sectional studies in male [7, 8], and after adjusted by gender [9]. A prospective study of civil servants showed effort-reward imbalance at work associated with alcohol dependence in male [10]. Limited cohort studies have been conducted with validated measurements for workplace strain and harmful alcohol use in the general working population [10]. Given this situation, identifying causal relationships between work-related stress and hazardous alcohol use could have high public health relevance.

## 2. Materials and methods

### 2.1 Data source and study population

#### 2.1.1 Internet survey

We used two data sets. One data is from the Japan Society and New Tobacco Internet Survey (JASTIS), a large internet-based cohort study that has been focusing on tobacco issues since 2015 and has expanded to various health behaviors since 2021. The other data is from the Japan COVID-19 and Society Internet Survey (JACSIS), a large internet-based cohort study that has been research about health disparity in Japan in various health aspects. The JASTIS and JACSIS cohort profile have been respectively published [11, 12]. JASTIS and JACSIS were open-cohort research, which followed same population and added new recruiting participants at each time, although each survey had different targeted questions.

The present study utilized two waves of longitudinal data from February 2021 (baseline, JASTIS2021) to February 2022 (JASTIS2022) and combined the data from October 2022 (JACSIS 2022) with user ID. These web-based, self-reported questionnaire surveys were administrated by a large internet research agency, Rakuten Insight, Inc., which pooled approximately 2.3 million panelists. Survey requests were sent by the research agency to the panelists, who were each selected based on gender, age, and prefecture. The panelists who consented to participate accessed the designated website and responded to the survey. The participants were given the option not to respond to any part of the survey or discontinue it at any point. The survey was closed when the target number of respondents for gender, age, and prefecture was met. The total number of participants in each survey was 33,000 and 34,000 participants in 2021 and 2022 in JASTIS, and 32,000 participants in 2022 in JACSIS, respectively.

#### 2.1.2 Managing the data quality and generating the study population

To ensure data quality, respondents with discrepancies or artificial/unnatural responses were excluded from the analysis based on the study protocol [11], where 1) If a respondent did not follow the question “Please choose the second item from the bottom,” 2) If a respondent answered ‘yes’ in all questions on alcohol and nine illegal drug use, and 3) If a respondent answered ‘yes’ in all questions regarding nine chronic diseases. Moreover, respondents who had inconsistent answers to alcohol-related questions were excluded from JASTIS: respondents who answered “Never” in the question “Do you drink alcohol?” but answered “monthly or more” in the question “How often do you have a drink containing alcohol?”. JACSIS contained Brief Job Stress Questionnaire (BJSQ). As the BJSQ targeted participants who regularly worked for income, students and retired people, who may work part-time, and full-time housemakers were excluded from this study. The data was divided by the AUDIT patterns change from 2022 to 2023, and this study focused on the population of non-problem drinking at 2022 to examine the change to problem drinking. Overall, the total number of follow-up participants was 8,157. The detailed selection flow of the study population is shown in Fig. 1.

**Fig. 1:**
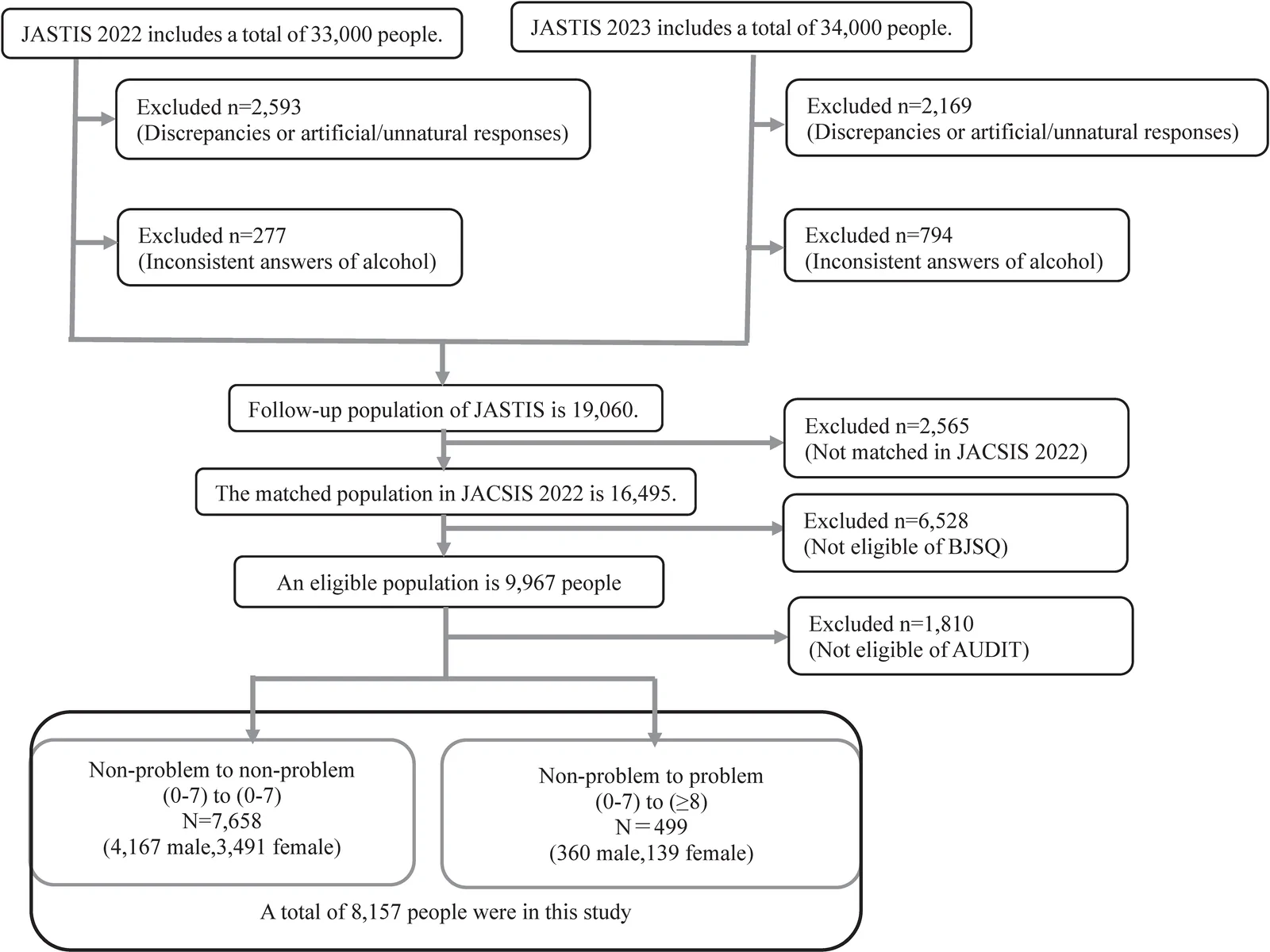
The flow diagram of the study population The figure shows the steps used in the selection of the members of this study. Information on the two cohort studies is available on the website: JASTIS (https://jastis-study.jp/), and JACSIS (https://jacsis-study.jp/index.html).

### 2.2 Measures

#### 2.2.1 Explanatory variables

In this study, the Brief Job Stress Questionnaire (BJSQ) was used as explanatory variables to assess work-related stress. The BJSQ have confirmed reliability and construct validity in other study [13]. The BJSQ has been widely implemented annually by the Ministry of Health, Labour and Welfare as a tool for measuring work-related stress, which amendments to the Industrial Safety and Health Act made mandatory for workplaces with 50 or more regular employees to conduct stress checks once a year. Items from BJSQ were included in JACSIS survey. The BJSQ consisted of 9 work-related stress categories such as demand from job quantity, demand from job quality, physical burden, relationship stress at workplace, working environmental stress, degree of job control, skills utilization, suitability for their job, and job satisfaction. Those scales were divided into three groups (low, medium, and high) based on the standard cut-off points in each category of work-related stress including gender-specific cut-offs were applied as per standard BJSQ scoring [14]. Details of each question and cut-off criteria were presented in Supplemental Table 1.

#### 2.2.2 Demographics and potential health factors related to alcohol use

The demographic questions were inquired about the following variables: age, gender, educational level, job, equivalent annual household income, marital status, and current living arrangements. Educational level was categorized as low (graduated from high school or lower), middle (graduated from vocational or junior college), and high (graduated from university or higher). Marital status was categorized as married, single, and divorced/widowed. The current living arrangements reported whether the participants lived with someone or alone. Jobs were categorized into regular jobs including executive/management level, permanent employee, self-employee, and non-permanent employees. Equivalent annual household income is the household income divided by the square root of the number of household members; the estimate was categorized as <2 million yen, 2–4 million yen, 4–6 million yen, 6–10 million yen, ≥10 million yen, and “do not know/want to answer”. Smoking status was categorized as never-, ex-, and current smokers and the subjective health condition was did as being poor, not good, fair, good, or excellent.

#### 2.2.3 Outcome variables

Nondrinkers and current drinkers were identified by the Alcohol Use Disorders Identification Test (AUDIT), which was developed by WHO and is one of the most effective screening tools in identifying individuals with alcohol use disorders and those involved in harmful alcohol use [15]. AUDIT is a 10-item screening measure that assesses alcohol use during the last 12 months [16, 17]. Although the AUDIT does not intend to offer a clinical diagnosis, it can indicate the presence and severity of alcohol problems or alcohol use disorders [18]. Furthermore, the AUDIT translated into Japanese was validated to identify harmful to hazardous alcohol use and alcohol use disorders as a screening tool [19]. The details of each question and score on each item are shown in Supplemental Table 2. The total scores range from 0 to 40 points. According to the National Health Guidance and Brief Intervention to Support Alcohol Reduction in Japan based on a validation study, an AUDIT score of 0–7 is considered normal, people with a score 8–14 are advised to reduce hazardous drinking, and people with a score ≥15 are recommended to see a specialist for further diagnostic evaluation of alcohol dependence [20]. The participants of this study were categorized into two groups based on the total score: non- problem drinking (AUDIT: 0–7) and problem drinking (AUDIT: 8–40) [16, 20, 21]. We used the established AUDIT cutoff of ≥8 to define problem drinking because our primary interest was the development of relevant alcohol-related risk, encompassing hazardous, harmful, and dependent drinking patterns.

Changes in AUDIT scores from 2022 to 2023 were categorized into four patterns: no changes from non-problem drinking (0–7) to non-problem drinking (0–7), non-problem drinking (0–7) to problem drinking (8–40), no changes from problem drinking (8–40) to problem drinking (8–40), and problem drinking (8–40) to non-problem drinking (0–7). Then, this study utilized two patterns: no changes from non-problem drinking to non-problem drinking and changed from non-problem drinking in 2022 to problem drinking in 2023.

### 2.3 Statistical analysis

Statistical analyses were performed using the Stata MP version 19 (Stata Corp). The means and standard deviations were presented as continuous variables and proportions for categorical variables. First, we determined variations in the means and proportions of demographics, and the potential health factors according to the AUDIT pattern change. Second, gender-specific multivariable binary logistic regression was performed among non-problem drinking at baseline to examine the association between job stress and the AUDIT patten change from non-problem drinking to problem drinking in reference to the pattern staining at non-problem drinking. Model 1 was adjusted for age, Model 2 was adjusted further for sociodemographic factors and potential phycological stress factors such as age, education, job, income, living alone, marital status [22], and smoking and subjective health. Model 3 was adjusted further for the job strains mutually. Age was continuous variable and other was categorical variable. Finally, the adjusted odds ratios (aORs) and 95% confidence intervals (CIs) for AUDIT pattern changes were calculated. In addition, the p for trend was tested using the median score of each job strain category. All statistical tests were two-sided, and a *p* value < 0.05 was considered statistically significant.

### 2.4 Ethical approval

All procedures were conducted as per the ethical standards of the 1975 Helsinki Declaration (revised in 2013). The Research Ethics Committee of the Tohoku University Graduate School of Medicine has reviewed and approved the study protocol (approval No.2024-1-1035). The Research Ethics Committee of the Kyushu University Graduate School of Medicine has approved the study protocol (approval No. 24179). All participants provided their informed consent before responding to the online questionnaire. Furthermore, the internet survey agency respected the Act on Protection of Personal Information in Japan. As an incentive, credit points (known as “E-points”), which can be used for internet shopping and cash conversion, were provided to participants.

## 3. Results

### 3.1 Participant characteristics

Of the 8,157 follow-up participants, 55% (n = 4,527) were men with a mean age of 47.1 (±13.9) years at baseline. Based on the participant characteristics, sociodemographic categories with the highest proportion of responses were 53% for high education level, 56% for married, 77% for living with someone, 46% for permanent employee, and 36% for income level of 2–4 million, 61% for never smokers and 34% for those with not good condition at subjective health (Table 1-1).

**Table 1-1:** Gender-specific baseline characteristics of participants according to the AUDIT pattern change

	**AUDIT pattern change from 2022 to 2023**				**Total**

	**Male**		**Female**		

	**Non-problem to ** **non-problem** **(0–7) to (0–7)**	**Non-problem to ** **problem drinking** **(0–7) to (≥8)**	**Non-problem to ** **non-problem** **(0–7) to (0–7)**	**Non-problem to ** **problem drinking** **(0–7) to (≥8)**	

	**N = 4167**	**N = 360**	**N = 3491**	**N = 139**	**N = 8157**

	**N (%)**	**N (%)**	**N (%)**	**N (%)**	**N (%)**
Age, year (Mean, SD)	47.1(13.9)	45.5(13.9)	45.4(14.2)	40.1(14.0)	47.2(14.0)
20–39 years	1314 (32)	131 (36)	1206 (35)	67 (48)	2718 (33)
40–59 years	1981 (48)	165 (46)	1649 (47)	58 (42)	3853 (47)
60–79 years	872 (21)	64 (18)	636 (18)	14 (10)	1586 (19)
Education					
Low	986 (24)	77 (21)	1004 (29)	20 (14)	2087 (26)
Middle	531 (13)	37 (10)	1081 (31)	44 (32)	1693 (21)
High	2630 (63)	244 (68)	1394 (40)	75 (54)	4343 (53)
Don’t want to answer	20 (<1)	2 (1)	12 (<1)	0 (0)	34 (<1)
Marital status					
Marriage	2570 (62)	247 (69)	1723 (49)	60 (43)	4600 (56)
No marriage	1390 (33)	92 (26)	1284 (37)	67 (48)	2833 (35)
Divorced/Widowed	207 (5)	21 (6)	484 (14)	12 (9)	724 (9)
Living alone	932 (22)	79 (22)	832 (24)	44 (32)	1887 (23)
Job					
Executive/management	853 (20)	101 (28)	203 (6)	5 (4)	1162 (14)
Permanent employee	2126 (51)	184 (51)	1375 (39)	73 (53)	3758 (46)
Self-employee	517 (12)	41 (11)	242 (7)	14 (10)	814 (10)
No-regular employee	671 (16)	34 (9)	1671 (48)	47 (34)	2423 (30)
Income					
Under 2 million yen	385 (9)	23 (6)	449 (13)	14 (10)	871 (11)
2–4 million yen	1520 (36)	149 (41)	1196 (34)	53 (38)	2918 (36)
4–6 million yen	969 (23)	84 (23)	613 (18)	30 (22)	1696 (21)
6–10 million yen	606 (15)	59 (16)	383 (11)	19 (14)	1067 (13)
10 million or more	110 (3)	14 (4)	69 (2)	2 (1)	195 (2)
Don’t want to answer	577 (14)	31 (9)	781 (22)	21 (15)	1410 (17)
Smoking					
Never-smoker	2040(49)	137(38)	2677(77)	93(67)	4947(61)
Past-smoker	1115(27)	123(34)	346(10)	14(10)	1598(20)
Current smoker	1012(24)	100(28)	468(13)	32(23)	1612(20)
Self-rated health condition					
Poor	930 (22)	84 (23)	765 (22)	31 (22)	1810 (22)
Not good	1419 (34)	127 (35)	1202 (34)	52 (37)	2800 (34)
Fair	1335 (32)	103 (29)	1075 (31)	44 (32)	2557 (31)
Good	397 (10)	37 (10)	376 (11)	10 (7)	820 (10)
Excellent	86 (2)	9 (2)	73 (2)	2 (1)	170 (2)

### 3.2 Gener-specific characteristics of participants according to AUDIT pattern change

Gender-specific characteristics of participants according to AUDIT pattern change from 2022 to 2023 are shown in Table 1-1. The number of participants who changed from non-problem drinking (AUDIT 0–7) to problem drinking (AUDIT ≥8) was 360 male and 139 female (6.1% of the follow-up population). The mean age (± SD) in who changed from non-problem to problem drinking was 45.5 (±13.9) years in male, and 40.1 (±14.0) years in female. The proportions of male participants who stated at non-problem drinking versus those who changed to problem drinking were such as 32% vs 36% for 20–39 years old, 63% vs 68% for higher education level, 62% vs 69% for married and 49% vs 38% never-smoker. Similar trends were observed in female. In addition, we examined the distribution of baseline AUDIT score according to work-related stress in 2022 (Supplemental Table 3). The people with higher AUDIT scores at baseline tended to have higher relationship stress, job control, and skill utilization for job, job satisfaction and lower physical demand and environmental stress.

The characteristic between work-related stress and the AUDIT pattern change from 2022 to 2023 are shown in Table 1-2. The proportions of male participants who stated at non-problem drinking versus those who changed to problem drinking were 25% vs 35% for high relationship stress, and 14% vs 19% for high job satisfaction. Similar trends in relationship stress were observed in female.

**Table 1-2:** Gender-specific characteristics of participants according to work-related stress and the AUDIT pattern change

	**AUDIT pattern change from 2022 to 2023**			

	**Male**		**Female**	

	**Non-problem to ** **non-problem** **(0–7) to (0–7)**	**Non-problem to ** **problem drinking** **(0–7) to (≥8)**	**Non-problem to ** **non-problem** **(0–7) to (0–7)**	**Non-problem to ** **problem drinking** **(0–7) to (≥8)**

	**N = 4167**	**N = 360**	**N = 3491**	**N = 139**

	**N (%)**	**N (%)**	**N (%)**	**N (%)**
Relationship stress				
Low (3–5)	1192 (29)	74 (21)	1286 (37)	45 (32)
Medium (6–7)	1919 (46)	159 (44)	1469 (42)	52 (37)
High (8–12)	1056 (25)	127 (35)	736 (21)	42 (30)
Demand (Quantity)				
Low (3–7) M/ (3–6) F	1984 (48)	140 (39)	1273 (36)	44 (32)
Medium (8–9) M/ (7–9) F	1434 (34)	139 (39)	1639 (47)	66 (47)
High (10–12)	749 (18)	81 (22)	579 (17)	29 (21)
Demand (Quality)				
Low (3–7) M/ (3–6) F	1535 (37)	123 (34)	980 (28)	38 (27)
Medium (8–9) M/ (7–8) F	1778 (43)	152 (42)	1219 (35)	44 (32)
High (10–12) M/ (9–12) F	854 (20)	85 (24)	1292 (37)	57 (41)
Physical demand				
Low (1)	1307 (31)	95 (26)	1200 (34)	47 (34)
Medium (2)	1332 (32)	124 (34)	917 (26)	34 (24)
High (3–4)	1528 (37)	141 (39)	1374 (39)	58 (42)
Environmental stress				
Low (1)	1076 (26)	91 (25)	908 (26)	30 (22)
Medium (2)	1731 (42)	135 (38)	1420 (41)	59 (42)
High (3–4)	1360 (33)	134 (37)	1163 (33)	50 (36)
Control				
Low (3–6) M/ (3–5) F	894 (21)	93 (26)	478 (14)	18 (13)
Medium (7–8) M/ (6–8) F	1117 (27)	93 (26)	1570 (45)	62 (45)
High (9–12)	2156 (52)	174 (48)	1443 (41)	59 (42)
Skills Utilization				
Low (1–2)	1416 (34)	118 (33)	1273 (36)	44 (32)
Medium (3)	1825 (44)	162 (45)	1526 (44)	69 (50)
High (4)	926 (22)	80 (22)	692 (20)	26 (19)
Suitability for the job				
Low (1–2)	1323 (32)	129 (36)	967 (28)	48 (35)
Medium (3)	2175 (52)	165 (46)	1863 (53)	63 (45)
High (4)	669 (16)	66 (18)	661 (19)	28 (20)
Job satisfaction				
Low (1–2)	1576 (38)	144 (40)	1209 (35)	57 (41)
Medium (3)	1991 (48)	149 (41)	1736 (50)	55 (40)
High (4)	600 (14)	67 (19)	546 (16)	27 (19)

### 3.3 Gender-specific associations between work-related stress and AUDIT pattern change from non-problem to problem drinking

Table 2 presents the gender-specific odds ratio (OR) of the AUDIT pattern change from non-problem to problem drinking according to work-related stress. In the ages adjusted model (Model 1), relationship stress at workplace was associated with the change from non-problem to problem drinking: the OR for high versus low relationship stress was 1.85 (95% CI: 1.36–2.52) in male, and 1.40 (95% CI: 0.91–2.16) in female. These associations between relationship stress at work and the increased risk of problem drinking pattern were a little attenuated in male and increased in female in Models 2 and 3. The adjusted OR in Model 3 was 1.77 (95% CI: 1.20–2.61) in male, and 1.74 (95% CI: 1.00–3.04) in female. The physical demand was marginally associated with the AUDIT pattern changein male (aOR in Model 3: 1.39, 95% CI: 1.00–1.94) but not in female.

**Table 2:** Gender-specific odds ratios of the AUDIT patten change according to each work-related stress

	**Non-problem drinking (AUDIT: 0–7) to problem drinking (AUDIT ≥8) N = 499**					

	**Male (N = 360)**			**Female (N = 139)**		

	**Model 1**	**Model 2**	**Model 3**	**Model 1**	**Model 2**	**Model 3**

	**OR (95%CI)**	**OR (95%CI)**	**OR (95%CI)**	**OR (95%CI)**	**OR (95%CI)**	**OR (95%CI)**
Relationship stress						
Low (3–5)	Ref*	Ref*	Ref*	Ref	Ref*	Ref*
Medium (6–7)	1.30(0.97, 1.73)	1.20(0.88, 1.64)	1.30(0.92, 1.84)	0.92(0.61, 1.38)	1.10(0.69, 1.75)	1.04(0.62, 1.73)
High (8–12)	**1.85(1.36, 2.52)**	**1.73(1.24, 2.42)**	**1.77(1.20, 2.61)**	1.40(0.91, 2.16)	**1.83(1.11, 3.00)**	**1.74(1.00, 3.04)**
Demand (Quantity)						
Low (3–7) M/ (3–6) F	Ref*	Ref*	Ref	Ref	Ref	Ref
Medium (8–9) M/ (7–9) F	1.32(1.03, 1.70)	1.20(0.92, 1.58)	1.16(0.86, 1.57)	1.05(0.71, 1.56)	1.26(0.81, 1.97)	1.36(0.81, 2.29)
High (10–12)	**1.47(1.10, 1.96)**	**1.39(1.02, 1.90)**	1.21(0.82, 1.77)	1.23(0.76, 2.00)	1.51(0.88, 2.59)	1.48(0.76, 2.91)
Demand (Quality)						
Low (3–7) M/ (3–6) F	Ref	Ref	Ref	Ref	Ref	Ref
Medium (8–9) M/ (7–8) F	1.06(0.83, 1.36)	0.96(0.74, 1.26)	0.85(0.63, 1.15)	0.93(0.60, 1.44)	0.78(0.48, 1.29)	0.67(0.39, 1.16)
High (10–12) M/ (9–12) F	1.22(0.91, 1.63)	1.16(0.86, 1.58)	0.86(0.59, 1.27)	1.11(0.73, 1.69)	1.05(0.66, 1.67)	0.77(0.43, 1.39)
Physical demand						
Low (1)	Ref	Ref*	Ref	Ref	Ref	Ref
Medium (2)	1.27(0.96, 1.68)	**1.38(1.02, 1.86)**	1.34(0.98, 1.85)	0.97(0.62, 1.53)	1.17(0.72, 1.92)	1.13(0.68, 1.88)
High (3–4)	1.23(0.93, 1.61)	**1.50(1.11, 2.03)**	**1.39(1.00, 1.94)**	1.08(0.73, 1.60)	1.30(0.83, 2.03)	1.19(0.73, 1.94)
Environmental stress						
Low (1)	Ref	Ref	Ref	Ref	Ref	Ref
Medium (2)	0.90(0.68, 1.19)	0.95(0.70, 1.29)	0.89(0.64, 1.24)	1.23(0.79, 1.93)	1.21(0.74, 1.97)	1.11(0.66, 1.87)
High (3–4)	1.11(0.84, 1.47)	1.17(0.86, 1.59)	0.97(0.69, 1.37)	1.20(0.76, 1.91)	1.21(0.73, 2.02)	1.05(0.61, 1.82)
Control						
Low (3–6) M/ (3–5) F	Ref	Ref*	Ref	Ref	Ref	Ref
Medium (7–8) M/ (6–8) F	0.81(0.60, 1.09)	0.76(0.55, 1.06)	0.80(0.57, 1.12)	1.09(0.64, 1.86)	1.10(0.62, 1.96)	1.21(0.66, 2.22)
High (9–12)	0.81(0.62, 1.05)	**0.71(0.52, 0.95)**	0.78(0.56, 1.09)	1.21(0.70, 2.08)	1.05(0.58, 1.90)	1.32(0.69, 2.52)
Skills Utilization						
Low (1–2)	Ref	Ref	Ref	Ref	Ref	Ref
Medium (3)	1.08(0.84, 1.38)	1.02(0.78, 1.33)	1.18(0.89, 1.55)	1.31(0.89, 1.93)	1.33(0.88, 2.03)	1.48(0.96, 2.28)
High (4)	1.08(0.80, 1.45)	1.00(0.72, 1.38)	1.12(0.78, 1.60)	1.10(0.67, 1.80)	0.88(0.49, 1.55)	0.96(0.52, 1.79)
Suitability for the job						
Low (1–2)	Ref	Ref	Ref	Ref	Ref	Ref
Medium (3)	0.81(0.63, 1.03)	0.72(0.55, 0.94)	0.80(0.58, 1.10)	0.77(0.52, 1.13)	0.69(0.45, 1.05)	0.80(0.49, 1.32)
High (4)	1.06(0.78, 1.45)	1.07(0.76, 1.51)	0.98(0.59, 1.61)	0.99(0.61, 1.60)	0.82(0.47, 1.42)	0.81(0.37, 1.78)
Job satisfaction						
Low (1–2)	Ref	Ref	Ref	Ref	Ref	Ref
Medium (3)	0.84(0.66, 1.07)	0.81(0.62, 1.05)	1.01(0.74, 1.39)	0.71(0.49, 1.04)	0.60(0.40, 0.92)	0.67(0.41, 1.09)
High (4)	1.27(0.93, 1.72)	1.33(0.95, 1.86)	1.61(0.98, 2.64)	1.12(0.70, 1.79)	0.99(0.59, 1.69)	1.21(0.57, 2.58)

Model 1: Adjusted for age (continuous variable)

Model 2: Adjusted further for socioeconomic factors (age, education, job, income, living alone, marital status) and health factors (smoking, subjective health)

Model 3: Adjusted further for job strains mutually

*p for trend < 0.05 (median), OR: odds ratio, 95%CI: 95% confidence interval

When an AUDIT score in 2022 was added in the multivariate model 3, the results did not mutually change (Supplemental Table 4). We also presented the gender-specific results and found the associations between relationship stress at work and the AUDIT patten change from non-problem to problem drinking did not vary between the genders (p for interaction = 0.58). Also, the gender interactions for other work-related stress were not statistically significant.

## 4. Discussion

In this nationwide longitudinal study, relationship stress at workplace and physical demand in male was associated with the increased risk of developing of problem alcohol drinking with 1-year follow-up, independent of subjective health conditions and other potential confounders. A similar but marginal association was observed for relationship stress in female.

According to previous cross-sectional analysis using Korean Longitudinal Study of Aging and the Japanese Study of Aging and Retirement, showed that work related stress was associated with the prevalence of heavy drinking. Specifically, effort-reward imbalance for job was associated with the prevalence of heavy drinking (210 g/week> in male, 140 g/week> in female) in Koreans but not in Japanese it was not associated with heavy drinking [9]. A Spanish study of 13,005 (male 60%) working individuals, aged 16–64 showed an associations of the lack of social support and noxious working environment such as dangerous work, hot or cold condition and unpleasant smell, noise or uncomfortable position with the prevalence of heavy drinking (≥40 g for male for daily averages) in male [8]. The Whitehall II study of 8,280 (male 69%) civil servants aged 35–55 in 1985–88 and followed up at 1991–93 showed that the effort-reward imbalance defined based on the combination of high effort scale by competitiveness, work related over commitment or hostility, and low rewards scale by poor promotion prospects or blocked career was associated with the risk of alcohol dependance, measured by validated CAGE questionnaire, in male but no association in female [10]. However, the measurements of effort-reward imbalance have not been standardized, so that the valid standardized measurement for effort-reward imbalance is necessary in further studies with multidimensionally constructed measures of job effort or reward.

Prior exposure to chronic harassment at workplace has been reported as a factor associated with alcohol misuse in 2,352 university employees (male 46%, average age: 39.9 years with 25 years follow-up) [23]. We assume that relationship stress may deteriorate and become chronic, potentially escalating into harassment. Such progression may partly explain the association between relationship stress and the risk of problem drinking.

Some professions characterized by the high physical demand such as construction workers may share their drinking habits as part of their occupational culture [24, 25], which may explain in part of the association between physical demand and problem drinking in our study. BSQJ questionnaire simply asked the physical demand “My job requires a lot of physical work”, but this measurement may reflect some specific professional work rather than office works.

The surveys were conducted during the recovery phase following the COVID-19 pandemic from 2022 to 2023, when changes in work arrangements, such as remote and hybrid work, may have influenced both occupational stress and drinking behaviors. Therefore, the observed associations may not fully reflect usual workplace conditions. Nevertheless, psychosocial work stress continues to be a relevant occupational health concern across diverse work arrangements, suggesting that the present findings may be useful for workplace health promotion in contemporary settings.

The main strength of this study was its large sample size of general working population to evaluate the association between work-related stress and the development of problem drinking, including probable alcohol dependence. Second, both self-reported work-related stress questionnaire based on the BJSQ [13] and drinking patterns based on the AUDIT are accepted as relevant screening tests and are reliable and valid in Japanese individuals [19, 26]. Third, strength point is that we examined the association with AUDIT patterns not only developing problem drinking but also maintaining problem drinking and mitigating problem drinking. In Japan, workplaces are legally required to conduct the BJSQ annually and health checkup including questions for drinking behavior. By leveraging BJSQ results, occupational health professionals can advise individuals showing elevated stress scores to targeted interventions during health checkups or workplace health programs. Relationship stress management may help reduce occupational stress and potentially contribute to reducing alcohol consumption and drinking opportunities.

However, this study also had several limitations. First, the current study design restricted the finding from being generalized to Japanese populations. Our data showed that 5% of people (7% for men and 2% for women) had an AUDIT score ≥15, which was greater than that reported in the national survey (2.9%; 5.2% for men and 2.9% for women) in 2018 [27]. Yet general full-time working population who have various job status and various geographical populations allowed us to examine the association between work-related stress and development of problem drinking among general working population. Second, although we adjusted for many potential confounders, the possibility of residual confounding, such as participants’ personality trait, cannot be excluded. Third, working-related stress was assessed in October 2022, eight months after baseline and four months before the second outcome measurement, which lacked the baseline work-related stress. Thus, the changes in these stress levels during the intervention period could influence the exposure classification. Forth, some mutual adjustment in Model 3 may have introduced multicollinearity, potentially inflating standard errors and attenuating effect estimates, because BJSQ domains are conceptually and empirically related to each other. However, the relationship stress remains associated with problem drinking in both men and women. Fifth, as participants were recruited through a commercial internet survey panel and received an honorarium for participation, the findings may be subject to selection bias and may not be fully generalizable to the broader working populations.

## 5. Conclusion

This study examined the association between work-related stress, more specifically relationship stress in workplace, and the increased risk of hazardous alcohol consumption patterns. The management of relationships at workplace, making a friendly atmosphere in the workplace within and beyond the department, may attenuate on the development of problem drinking.

## Abbreviations

AUDIT: Alcohol Use Disorders Identification Test
aOR: Adjusted odds ratios
BJSQ: The New Brief Job Stress Questionnaire
CI: Confidence interval
JACSIS: The Japan COVID-19 and Society Internet Survey
JASTIS: Japan Society and New Tobacco Internet Survey
SD: Standard deviation
WHO: World Health Organization

## Supplementary information

**Additional file 1:** **Supplemental Table 1.** The scale score of our study based on the Brief Job Stress Questionnaire (BJSQ). **Supplemental Table 2.** AUDIT questions and each score of answers. **Supplemental Table 3.** Baseline AUDIT score according to work-related stress in 2022. **Supplemental Table 4.** Gender-interaction for associations between work-related stress and AUDIT pattern change, adjusted by 2022 AUDIT score.

## Declarations

### Data availability statement

The data that support the findings of this study are available on reasonable request. However, restrictions apply to the availability of these data to protect personal identification; research data are not shared. If any person wishes to verify our data, they are welcome to contact the corresponding author.
