# Supplementary material for: Relationship stress at workplace was associated with problem drinking patterns: a nationwide Internet-based prospective cohort study
Source: Environ Health Prev Med. 2026 Sep 19;31:63. doi: 10.1265/ehpm.26-00131 (PMC13617186; doi:10.1265/ehpm.26-00131)
Supplement: Supplementary file 1 [file ehpm-31-063-s001.docx]

Supplemental Table 1. The scale score of our study based on the Brief Job Stress Questionnaire (BJSQ)

|  | The scale score in our study based on BJSQ | | | |
| --- | --- | --- | --- | --- |
|  | Very much | Moderately | Somewhat | Not at all |
| Demand (Quantity) | | | | |
| 1. I have an extremely large amount of work to do | 4 | 3 | 2 | 1 |
| 2. I can't complete work in the required time | 4 | 3 | 2 | 1 |
| 3. I have to work as hard as I can | 4 | 3 | 2 | 1 |
| Demand (Quality) | | | | |
| 4. . I have to pay very careful attention | 4 | 3 | 2 | 1 |
| 5. My job is difficult in that it requires a high level of knowledge and technical skill | 4 | 3 | 2 | 1 |
| 6. I need to be constantly thinking about work throughout the working day | 4 | 3 | 2 | 1 |
| Physical demand | | | | |
| 7. My job requires a lot of physical work | 4 | 3 | 2 | 1 |
| Job control | | | | |
| 8. I can work at my own pace | 4 | 3 | 2 | 1 |
| 9. I can choose how and in what order to do my work | 4 | 3 | 2 | 1 |
| 10. I can reflect my opinions on workplace policy | 4 | 3 | 2 | 1 |
| Skills Utilization | | | | |
| 11. My knowledge and skills are rarely used at work | 1 | 2 | 3 | 4 |
| Relationship stress at workplace | | | | |
| 12. There are differences of opinion within my department | 4 | 3 | 2 | 1 |
| 13. My department does not get along well with other departments | 4 | 3 | 2 | 1 |
| 14. The atmosphere in my workplace is friendly | 1 | 2 | 3 | 4 |
| Environmental stress | | | | |
| 15. My working environment is poor (e.g. noise, lighting, temperature, ventilation) | 4 | 3 | 2 | 1 |
| Suitability for the job | | | | |
| 16. This job suits me well | 4 | 3 | 2 | 1 |
| Job satisfaction | | | | |
| 17. My job is worth doing | 4 | 3 | 2 | 1 |

*The cut off point is deferent among the categories. The demand (Quantity), demand (Quality) and job control have different cut off point between man and women. The cut off points show in the Table3. The details of reliability and validity was shown the “Inoue A. et al. (2014)” on the reference list.

Supplemental Table 2. AUDIT questions and each score of answers

|  | 0 | 1 | 2 | 3 | 4 |
| --- | --- | --- | --- | --- | --- |
| 1. How often do you have a drink containing alcohol? | Never | Monthly or less | 2 to 4 times a month | 2 to 3 times a week | 4 or more times a week |
| 2. How many drinks containing alcohol do you have on a typical day when you are drinking? | 1 or 2 | 3 or 4 | 5 or 6 | 7, 8, or 9 | 10 or more |
| 3. How often do you have six or more drinks on one occasion? | Never | Less than monthly | Monthly | Weekly | Daily or almost daily |
| 4. How often during the last year have you found that you were not able to stop drinking once you started? | Never | Less than monthly | Monthly | Weekly | Daily or almost daily |
| 5. How often during the last year have you failed to do what was normally expected from you because of drinking? | Never | Less than monthly | Monthly | Weekly | Daily or almost daily |
| 6. How often during the last year have you needed a first drink in the morning to get yourself going after a heavy drinking session? | Never | Less than monthly | Monthly | Weekly | Daily or almost daily |
| 7. How often during the last year have you had a feeling of guilt or remorse after drinking? | Never | Less than monthly | Monthly | Weekly | Daily or almost daily |
| 8. How often during the last year have you been unable to remember what happened the night before because you had been drinking? | Never | Less than monthly | Monthly | Weekly | Daily or almost daily |
| 9. Have you or someone else been injured as a result of your drinking? | No |  | Yes, but not in the last year |  | Yes, during the last year |
| 10. Has a relative or friend or a doctor or another health worker been concerned about your drinking or suggested you cut down? | No |  | Yes, but not in the last year |  | Yes, during the last year |

| Supplemental Table 3. Baseline AUDIT score according to work-related stress in 2022 | | | | | | | | | | |
| --- | --- | --- | --- | --- | --- | --- | --- | --- | --- | --- |
|  | AUDIT | | | | | | | | | |
|  | Total | 0 | 1 | 2 | 3 | 4 | 5 | 6 | 7 |  |
|  | N＝8,157 | N=2,702 | N=1,296 | N=1,028 | N=911 | N=898 | N=601 | N=398 | N=322 | p for trend |
| Relationship stress | N (%) | N (%) | N (%) | N (%) | N (%) | N (%) | N (%) | N (%) | N (%) |  |
| Low (3-5) | 2597 (32) | 843 (31) | 397 (31) | 324 (32) | 289 (32) | 294 (33) | 217 (36) | 129 (32) | 104 (32) | 0.045 |
| Medium (6-7) | 3599 (44) | 1197 (44) | 577 (45) | 448 (44) | 396 (43) | 401 (45) | 263 (44) | 182 (46) | 135 (42) |  |
| High (8-12) | 1961 (24) | 662 (25) | 322 (25) | 256 (25) | 226 (25) | 203 (23) | 121 (20) | 87 (22) | 84 (26) |  |
| Demand (Quantity) |  |  |  |  |  |  |  |  |  |  |
| Low (3-7) M/ (3-6) F | 3441 (42) | 1134 (42) | 518 (40) | 410 (40) | 373 (41) | 413 (46) | 259 (43) | 183 (46) | 151 (47) | 0.078 |
| Medium (8-9) M/ (7-9) F | 3278 (40) | 1116 (41) | 531 (41) | 425 (41) | 364 (40) | 337 (38) | 251 (42) | 139 (35) | 115 (36) |  |
| High (10-12) | 1438 (18) | 452 (17) | 247 (19) | 193 (19) | 174 (19) | 148 (16) | 91 (15) | 76 (19) | 57 (18) |  |
| Demand (Quality) |  |  |  |  |  |  |  |  |  |  |
| Low (3-7) M/ (3-6) F | 2676 (33) | 911 (34) | 403 (31) | 317 (31) | 298 (33) | 309 (34) | 198 (33) | 135 (34) | 105 (33) | 0.282 |
| Medium (8-9) M/ (7-8) F | 3193 (39) | 1004 (37) | 538 (42) | 403 (39) | 361 (40) | 358 (40) | 248 (41) | 155 (39) | 126 (39) |  |
| High (10-12) M/ (9-12) F | 2288 (28) | 787 (29) | 355 (27) | 308 (30) | 252 (28) | 231 (26) | 155 (26) | 108 (27) | 92 (28) |  |
| Physical demand |  |  |  |  |  |  |  |  |  |  |
| Low (1) | 2649 (32) | 815 (30) | 424 (33) | 337 (33) | 315 (35) | 304 (34) | 212 (35) | 116 (29) | 126 (39) | <0.001 |
| Medium (2) | 2407 (30) | 740 (27) | 392 (30) | 305 (30) | 270 (30) | 274 (31) | 193 (32) | 129 (32) | 104 (32) |  |
| High (3-4) | 3101 (38) | 1147 (42) | 480 (37) | 386 (38) | 326 (36) | 320 (36) | 196 (33) | 153 (38) | 93 (29) |  |
| Environmental stress |  |  |  |  |  |  |  |  |  |  |
| Low (1) | 2105 (26) | 696 (26) | 320 (25) | 264 (26) | 223 (24) | 250 (28) | 157 (26) | 101 (25) | 94 (29) | 0.024 |
| Medium (2) | 3345 (41) | 1076 (40) | 529 (41) | 433 (42) | 366 (40) | 377 (42) | 270 (45) | 161 (40) | 133 (41) |  |
| High (3-4) | 2707 (33) | 930 (34) | 447 (34) | 331 (32) | 322 (35) | 271 (30) | 174 (29) | 136 (34) | 96 (30) |  |
| Control |  |  |  |  |  |  |  |  |  |  |
| Low (3-6) M/ (3-5) F | 1483 (18) | 550 (20) | 233 (18) | 179 (17) | 159 (17) | 155 (17) | 96 (16) | 58 (15) | 53 (16) | <0.001 |
| Medium (7-8) M/ (6-8) F | 2842 (35) | 986 (36) | 487 (38) | 368 (36) | 335 (37) | 274 (31) | 180 (30) | 128 (32) | 84 (26) |  |
| High (9-12) | 3832 (47) | 1166 (43) | 576 (44) | 481 (47) | 417 (46) | 469 (52) | 325 (54) | 212 (53) | 186 (58) |  |
| Skills Utilization |  |  |  |  |  |  |  |  |  |  |
| Low (1-2) | 2851 (35) | 974 (36) | 478 (37) | 326 (32) | 312 (34) | 312 (35) | 207 (34) | 137 (34) | 105 (33) | 0.049 |
| Medium (3) | 3582 (44) | 1179 (44) | 569 (44) | 459 (45) | 392 (43) | 398 (44) | 267 (44) | 179 (45) | 139 (43) |  |
| High (4) | 1724 (21) | 549 (20) | 249 (19) | 243 (24) | 207 (23) | 188 (21) | 127 (21) | 82 (21) | 79 (24) |  |
| Suitability for the job |  |  |  |  |  |  |  |  |  |  |
| Low (1-2) | 2467 (30) | 843 (31) | 399 (31) | 301 (29) | 286 (31) | 258 (29) | 181 (30) | 115 (29) | 84 (26) | 0.176 |
| Medium (3) | 4266 (52) | 1363 (50) | 698 (54) | 545 (53) | 469 (51) | 482 (54) | 323 (54) | 216 (54) | 170 (53) |  |
| High (4) | 1424 (17) | 496 (18) | 199 (15) | 182 (18) | 156 (17) | 158 (18) | 97 (16) | 67 (17) | 69 (21) |  |
| Job satisfaction |  |  |  |  |  |  |  |  |  |  |
| Low (1-2) | 2986 (37) | 1052 (39) | 486 (38) | 367 (36) | 321 (35) | 307 (34) | 203 (34) | 134 (34) | 116 (36) | 0.006 |
| Medium (3) | 3931 (48) | 1228 (45) | 640 (49) | 502 (49) | 448 (49) | 455 (51) | 309 (51) | 201 (51) | 148 (46) |  |
| High (4) | 1240 (15) | 422 (16) | 170 (13) | 159 (15) | 142 (16) | 136 (15) | 89 (15) | 63 (16) | 59 (18) |  |

P for trend was calculated to assess the linear trend between work-related stress and AUDIT scores ranging from 0 to 7.

Supplemental Table 4. Gender-interaction for association between work-related stress and AUDIT pattern change, adjusted by 2022 AUDIT score

|  | Non-problem drinking to Problem drinking | | | | | |  |
| --- | --- | --- | --- | --- | --- | --- | --- |
|  | Total | | Female | | Male | | *P for sex interaction |
| Relationship stress | OR | (95%CI) | OR | (95%CI) | OR | (95%CI) | 0.58 |
| Low (3-5) | ref |  | ref |  | ref |  |  |
| Medium (6-7) | 1.27 | (0.94, 1.72) | 1.20 | (0.69, 2.11) | 1.37 | (0.95, 1.97) |  |
| High (8-12) | **1.93** | **(1.37, 2.71)** | **2.12** | **(1.13, 3.97)** | **1.96** | **(1.30, 2.96)** |  |
| Demand (Quantity) |  |  |  |  |  |  | 0.72 |
| Low (3-7) M/ (3-6) F | ref |  | ref |  | ref |  |  |
| Medium (8-9) M/ (7-9) F | 1.26 | (0.96, 1.65) | 1.42 | (0.81, 2.49) | 1.30 | (0.94, 1.79) |  |
| High (10-12) | 1.37 | (0.96, 1.95) | 1.65 | (0.79, 3.42) | 1.39 | (0.92, 2.09) |  |
| Demand (Quality) |  |  |  |  |  |  | 0.79 |
| Low (3-7) M/ (3-6) F | ref |  | ref |  | ref |  |  |
| Medium (8-9) M/ (7-8) F | 0.77 | (0.59, 1.02) | 0.65 | (0.36, 1.18) | 0.80 | (0.59, 1.11) |  |
| High (10-12) M/ (9-12) F | 0.70 | (0.50, 0.99) | 0.66 | (0.34, 1.27) | 0.71 | (0.47, 1.06) |  |
| Physical demand |  |  |  |  |  |  | 0.16 |
| Low (1) | ref |  | ref |  | ref |  |  |
| Medium (2) | 1.22 | (0.92, 1.62) | 1.29 | (0.74, 2.24) | 1.26 | (0.89, 1.76) |  |
| High (3-4) | **1.44** | **(1.08, 1.92)** | 1.56 | (0.91, 2.68) | **1.48** | **(1.04, 2.10)** |  |
| Environmental stress |  |  |  |  |  |  | 0.94 |
| Low (1) | ref |  | ref |  | ref |  |  |
| Medium (2) | 0.93 | (0.69, 1.25) | 0.99 | (0.56, 1.75) | 0.89 | (0.62, 1.26) |  |
| High (3-4) | 0.98 | (0.72, 1.34) | 1.04 | (0.57, 1.90) | 0.96 | (0.66, 1.39) |  |
| Control |  |  |  |  |  |  | 0.85 |
| Low (3-6) M/ (3-5) F | ref |  | ref |  | ref |  |  |
| Medium (7-8) M/ (6-8) F | 0.82 | (0.60, 1.11) | 1.37 | (0.69, 2.70) | 0.74 | (0.52, 1.06) |  |
| High (9-12) | 0.75 | (0.55, 1.02) | 1.15 | (0.55, 2.39) | **0.68** | **(0.48, 0.96)** |  |
| Skills Utilization |  |  |  |  |  |  | 0.79 |
| Low (1-2) | ref |  | ref |  | ref |  |  |
| Medium (3) | 1.35 | (1.05, 1.73) | 1.51 | (0.93, 2.47) | 1.27 | (0.94, 1.70) |  |
| High (4) | 1.17 | (0.85, 1.63) | 1.24 | (0.63, 2.46) | 1.19 | (0.82, 1.73) |  |
| Suitability for the job |  |  |  |  |  |  | 0.37 |
| Low (1-2) | ref |  | ref |  | ref |  |  |
| Medium (3) | 0.83 | (0.63, 1.11) | 0.85 | (0.49, 1.47) | 0.82 | (0.59, 1.15) |  |
| High (4) | 1.01 | (0.65, 1.57) | 1.10 | (0.47, 2.58) | 0.97 | (0.57, 1.65) |  |
| Job satisfaction |  |  |  |  |  |  | 0.08 |
| Low (1-2) | ref |  | ref |  | ref |  |  |
| Medium (3) | 0.89 | (0.67, 1.17) | **0.44** | **(0.26, 0.77)** | 1.08 | (0.78, 1.51) |  |
| High (4) | 1.44 | (0.93, 2.22) | 0.64 | (0.28, 1.48) | **1.78** | **(1.05, 3.03)** |  |

OR with p<.05 shown in the bold letter.
